# 

**Published:** 1890-04

**Authors:** 


### BOOKS AND PAMPHLETS RECEIVED.

"Annual Report of the Canadian Institute," session 1889–90. Toronto, 1889.

"Twenty-fourth Annual Report American Society for the Prevention of Cruelty to Animals." New York, 1889.

"List of Accessions to the U. S. National Museum," during the year ending June 30th, 1886, with descriptive notes. Washington, 1889.

"Description of a new species of Etutænia," by Arthur Erwin Brown. Reprinted from Proceedings of the Academy of Natural Sciences of Philadelphia, 1890.

"Los Caballos Fosiles de la Pampa Argentina." Descriptos por Dr. German Burmeister, Director del Museo Nacional de Buenos Ayres, 1889.

"Bollettino Scientifico." Pavia, 1890.

"Il Naturalista Siciliano." Palermo, 1890.

"Archives Neerlandaises des Sciences. Exactes et Naturelles." Tome XXIV., Liv. I. Harlem, 1890.

"Sitzungs Berichte der Gesellschaft Naturforschender Freunde zu Berlin," 1889.

"Verhandlungen und Mittheilungen des Siebenburgischen Vereins fur Naturwissenschaften in Hermannstadt." XXXIX Jahr., 1889.

"Bulletin de L'Academie Royale de Médecine de Belgique." Brussels, 1890.

"Bulletin Mensuel Société Linneeune du Nord de la France." Amiens, 1890.

"Bulletin de la Société Zoologique de France." Paris, 1890.

"Annales de la Sociedad Cientifica. Argentina." Tomo XXVIII. Ent. 3–4. Buenos Ayres.

"Verhandlungen der K. K. Zoologisch—botanischen Gesellschaft." Bd. XXIX. Qu. 3–4. Vienna.

"Transactions Kansas Academy of Science." Vols. X–XI. Topeka, '85–'88.

"Johns Hopkins University Circular." March. Baltimore, 1890.

---

DEPARTMENT OF AGRICULTURE.—*Report of Statistician*, No. 70.—Numbers and Values of Farm Animals. T. R. Dodge, Jan. and Feb., 1890.

*Division of Chemistry*, No. 25.—Extent and Character of "Food Adulteration." Alex. J. Wedderburn, 1890. Contains interesting matter on the necessity of inspection of our meat foods, both live animals slaughtered for food and canned meat, and it shows that it is the poor who suffer most from want of inspection.

*Fourth and Fifth Annual Reports of Bureau of Animal Industry*, 1887–1888.

*Division of Entomology*, No. 21, 1890.—Natural Enemies of the Fluted Scale. Albert Koebele.

